# Dynamic Spatiotemporal Trends of Dengue Transmission in the Asia-Pacific Region, 1955–2004

**DOI:** 10.1371/journal.pone.0089440

**Published:** 2014-02-24

**Authors:** Shahera Banu, Wenbiao Hu, Yuming Guo, Suchithra Naish, Shilu Tong

**Affiliations:** 1 School of Public Health and Social Work, Queensland University of Technology, Brisbane, Australia; 2 School of Population Health, University of Queensland, Brisbane, Australia; University of California Davis, United States of America

## Abstract

**Background:**

Dengue fever (DF) is one of the most important emerging arboviral human diseases. Globally, DF incidence has increased by 30-fold over the last fifty years, and the geographic range of the virus and its vectors has expanded. The disease is now endemic in more than 120 countries in tropical and subtropical parts of the world. This study examines the spatiotemporal trends of DF transmission in the Asia-Pacific region over a 50-year period, and identified the disease’s cluster areas.

**Methodology and Findings:**

The World Health Organization’s DengueNet provided the annual number of DF cases in 16 countries in the Asia-Pacific region for the period 1955 to 2004. This fifty-year dataset was divided into five ten-year periods as the basis for the investigation of DF transmission trends. Space-time cluster analyses were conducted using scan statistics to detect the disease clusters. This study shows an increasing trend in the spatiotemporal distribution of DF in the Asia-Pacific region over the study period. Thailand, Vietnam, Laos, Singapore and Malaysia are identified as the most likely clusters (relative risk = 13.02) of DF transmission in this region in the period studied (1995 to 2004). The study also indicates that, for the most part, DF transmission has expanded southwards in the region.

**Conclusions:**

This information will lead to the improvement of DF prevention and control strategies in the Asia-Pacific region by prioritizing control efforts and directing them where they are most needed.

## Introduction

Dengue fever (DF) is one of the most important emerging arboviral diseases, and is widespread in tropical and subtropical parts of the world. It is estimated that approximately 3.6 billion people worldwide, and approximately 120 million travelers, are at risk of contracting the disease. There are approximately 50–100 million DF cases annually, and the mortality rate is approximately 2.5% [1]–[3]. The incidence of DF has increased 30-fold over the last fifty years, and the geographic range of the virus and its vectors has expanded [4]. Prior to 1970, only nine countries experienced DF epidemics; however, the disease is now endemic in more than 120 countries in Africa, America, the Eastern Mediterranean, South-east Asia and the Western Pacific [3]. Between 2000 and 2007, at least eight previously DF-free countries became infected; for example, suspected outbreaks were recorded in Pakistan, Saudi Arabia, Yemen, Sudan and Madagascar between 2005 and 2006 [5].

In Asia, epidemic DF was common during the first half of the 20^th^ century [6], and severe epidemics first occurred in the Philippines and Thailand during the 1950s. The recent geographic distribution of DF shows that the disease has now spread from Southeast Asian countries west to India, Sri Lanka, the Maldives and east to China. Several Pacific Island nations – such as the Cook Islands, Tahiti, New Caledonia, Vanuatu, Niue, and Palau – have also experienced DF outbreaks [7]. Nearly 1.8 billion people living in the Asia-Pacific region are currently at risk; indeed, this risk accounts for 70% of the global DF risk [3]. There are a number of reasons for the region’s high vulnerability to DF activity: the tropical climate of the region is suitable for DF transmission; there are four dengue viruses in the region; and the region has a high population density [8].

Geographic information systems (GIS) have been widely used in vector borne disease epidemiology. In disease mapping, such systems can visualize the spatiotemporal pattern and variation in disease risk. Monitoring the spatiotemporal trends in disease occurrence can highlight the changing patterns in risk and help to identify risk factors [9]. The spatial scan statistic is one of the most commonly used approaches in spatial disease surveillance to explore high-risk areas or disease clusters [10], [11]. The method scans a larger encompassing area for possible disease clusters, without a priori specification of their location and size. It identifies the approximate location of clusters and performs significance tests for each [11], [12]. The scan statistic is widely used because i) it adjusts for both inhomogeneous population density and various confounding factors; ii) it searches for clusters without the need to specify their size and location (This ameliorates the problem of pre-selection bias); iii) the likelihood ratio-based test statistics take multiple testing into account and give a single *p* value for the testing of the null hypothesis; and iv) on rejection of a null hypothesis, it is possible to specify the approximate location of the cluster that caused the rejection [11], [12].

GIS and spatial analyses have been used to identify geographic patterns and risk factors for DF transmission in various areas [13]–[19]. However, its spatial pattern remains unexplored at the continental level. This study addresses this deficit by examining the spatiotemporal patterns of DF in the Asia-Pacific region during the period 1955–2004, and identifying DF clusters in different periods. Such information is essential for improving DF prevention and control strategies.

## Methods

### Study Area

The continents of Australasia (Oceania and Asia) were selected as the study area because the Asian and Pacific regions are the most seriously affected by DF. With approximately 3.9 billion people, Asia is the largest and most populous continent in the world. The continent is located in the eastern and northern hemispheres and covers 44 579 000 km^2^ of the Earth’s surface. Its climate is moist across the southeast and dry across much of the interior. Because of the Himalayas, the monsoon circulation dominates the southern and eastern regions. This leads to the formation of a thermal low, which draws in moisture during the summer. South-Western parts of the continent are hot. The continent of Oceania includes Australia, New Zealand and a number of widely scattered island nations across the Pacific Ocean. Its total land area is 8 536 716 km^2^, with a population of 37 million. The islands of Oceania have a tropical or subtropical climate, which ranges from humid to seasonally dry.

### Data Collection

DengueNet data query, managed by the World Health Organization (WHO) [21], provided the annual number of DF cases for 16 countries of the Asia-Pacific region. DengueNet is an internet- based surveillance tool, which was established in 2005 to collect and provide current global DF epidemiological data and trends. Currently, it provides DF statistics from 1955 onwards. However, many countries did not report their DF outbreaks to the WHO during the period 2005–2012. For this reason, the study was restricted to the period 1955–2004. Of the 82 countries of the Asia-Pacific region, 22 countries reported DF outbreaks to the WHO during this period; however, only 16 of these countries were included in our analyses because the remainder did not report their known outbreaks to the WHO for more than five years during this period either.

The retrieved dataset for each country was compared with historic DF data (published in the literature) to check for data consistency. Location information, including coordinates, area and population size were collected from the Central Intelligence Agency (CIA) World Factbook [20]. The number of population censuses varies from country to country. Therefore, we chose two census periods for each country: the one closest to the beginning of our study period, and another towards the end of that period. The population size for the period before the first census was set as equal to the population size at the first census, while the population for the period after the last census was set as equal to the population at the last census. Linear interpolation was then used to estimate the population for the periods between censuses.

### Data Analyses

To investigate the spatial and temporal patterns of DF transmission, the fifty-year dataset was divided into five ten-year periods: A) 1955–1964; B) 1965–1974; C) 1975–1984; D) 1985–1994; and E) 1995–2004. Cumulative incidence rates for each period were mapped to visualize DF’s temporal trends. To calculate the cumulative incidence for each country, the annual DF incidence was first calculated by dividing the number of annual DF cases by the corresponding population and then multiplying by 100 000. These annual DF incidences were then aggregated for each ten-year period to estimate the cumulative incidence.

A “disease cluster” is an unusually high concentration of disease in a region, which is unlikely to occur by chance. Kulldorff’s space-time scan statistic (SaTScan) [11] was used to test for the presence of DF. In the analyses, it was assumed that the number of DF cases in each country was Poisson distributed. Then the null hypothesis that the number of cases is randomly distributed in geographic space and time, and that the expected cases in each area are proportional to its population [11], [12] was tested. The space-time scan statistic is defined by a cylindrical window with a circular geographic base, and height corresponding to time. This window is then moved in space and time to obtain an infinite number of overlapping cylinders of different sizes and shapes. Together, these cylinders cover the entire study region, and each reflects a possible cluster [11], [12].

The scan statistic tests the null hypothesis for each cylindrical window against the alternative hypothesis that there is an elevated risk of DF within the window, compared to outside the window [11]. SaTScan detects potential clusters by calculating a maximum likelihood ratio for each window [11]. The window with the maximum likelihood ratio is considered the most likely cluster. SaTScan also detects secondary clusters that have a significantly large likelihood ratio, but are not the most likely. To evaluate the statistical significance of both most likely and secondary clusters, SaTScan generates a large number of random replications of the dataset under the null hypothesis to obtain the *p*-value through Monte Carlo hypothesis testing. It then compares the rank of the maximum likelihood from the real dataset with the maximum likelihood from the random dataset [12]. In these analyses, 9999 Monte Carlo replications were used.

For cluster specification in space-time analyses, two parameters for the maximum cluster size were set: the proportion for the population at risk, and the proportion for the study period. The population density in the study area (16 countries) varies greatly, as does disease surveillance. Furthermore, due to the higher population density, more cases are usually expected in urban areas than in similar sized rural areas. To adjust for this uneven population density, and consistent with the previous literature relating to mosquito-borne diseases, it was decided to limit the spatial cluster size to 15% of the population at risk [21], [22]. However, analyses were conducted with maximum spatial cluster sizes of 50%, 40%, 30% and 20% of the population at risk to avoid pre-selection bias. The results were very similar to those obtained for the 15% population limit. A maximum of 50% of the study period was used as a maximum cluster size in the temporal window.

SaTScan software (Version 9.1.1) was used for the space-time scan statistic test [11], and R software (Version 2.12.0: R Development Core Team 2009) mapped all results. The R “maptools” package was used to translate the space-time outputs into maps and to visualize the DF clusters.

## Results

### Descriptive Statistics

**Figure 1:**
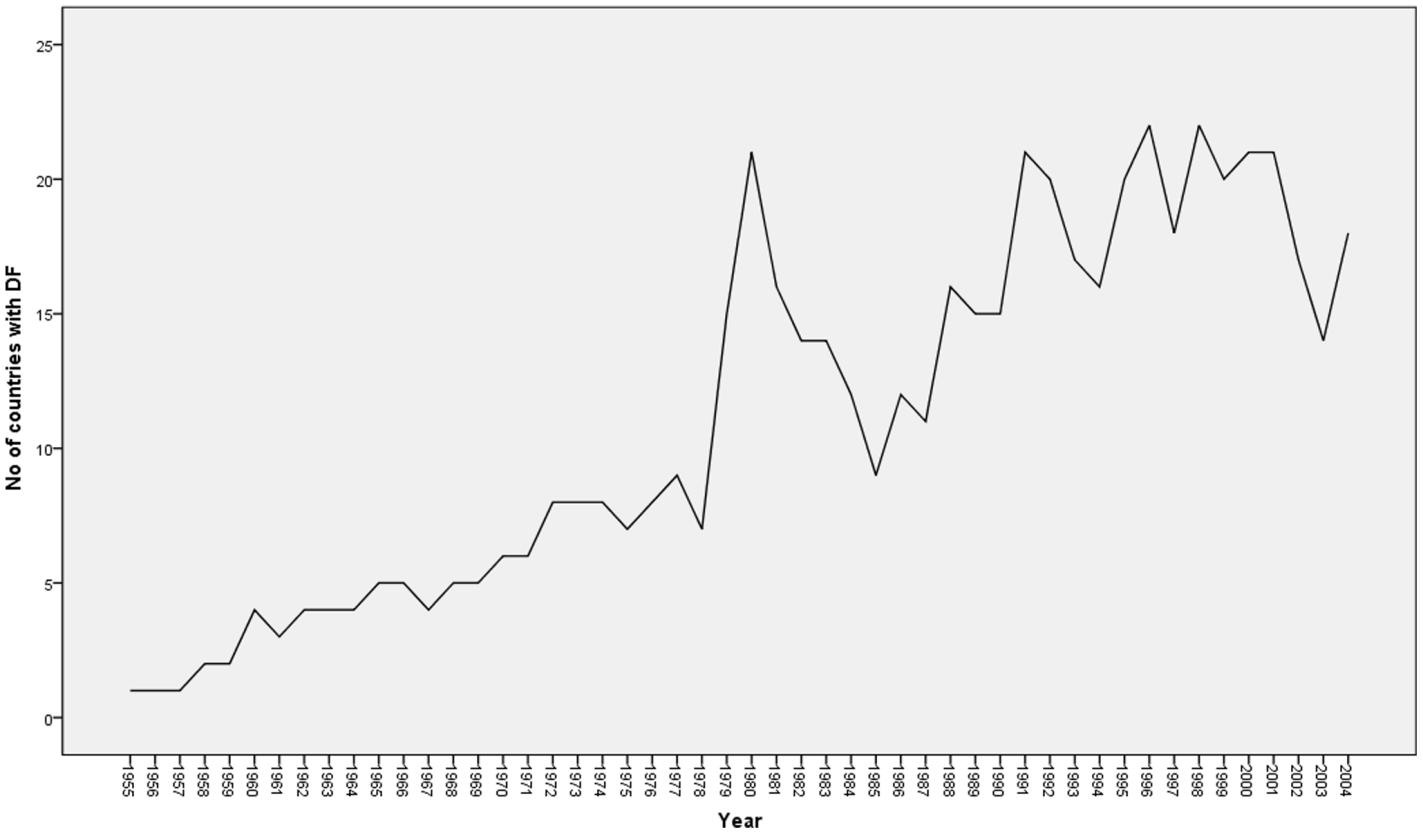
Total number of countries with DF outbreaks in the Asia-Pacific region, 1955–2004 (Data source: WHO DengueNet).

The annual number of DF cases for the selected countries ranged from 0 to 3 54 517 during the study period (1995–2004) (See Table 1). The lowest average number of cases was reported in Tuvalu (17), and the highest in Vietnam (41 819). The number of countries affected by DF dramatically increased over time (Figure 1), and 22 (26%) of the Asia-Pacific countries reported at least one DF outbreak in these fifty years.

**Table 1:** Annual number of DF cases in Asian-Pacific countries, 1955–2004.

Countries (N = 16)	Minimum	25%	Median	75%	Maximum	Mean	Std. Deviation
Australia	0	0	0	44	868	88	189
Bangladesh	0	0	0	0	6,104	378	1,300
Cook Islands	0	0	0	25	2,256	126	437
India	0	0	0	773	16,517	1,552	3,609
Indonesia	0	0	6,449	21,552	78,690	14,948	19,258
Laos	0	0	0	1,733	17,690	1,553	3315
Malaysia	0	0	810	5,508	33,895	4,932	9,002
Maldives	0	0	0	0	2,054	99	388
Micronesia	0	0	0	0	700	30	134
Myanmar	0	0	1,795	4,854	16,047	3,177	4,053
Philippines	0	388	1,042	6,342	35,648	4,985	8,236
Singapore	0	91	273	1,268	9,459	1,105	1,797
Sri Lanka	0	0	1	679	15,408	942	2,621
Thailand	0	5,914	23,018	45,555	1,74,285	33,814	38,637
Tuvalu	0	0	0	0	811	17	114
Vietnam	0	40	27,306	49,668	3,54,517	41,819	63,532

### Trends of DF Transmission

**Figure 2:**
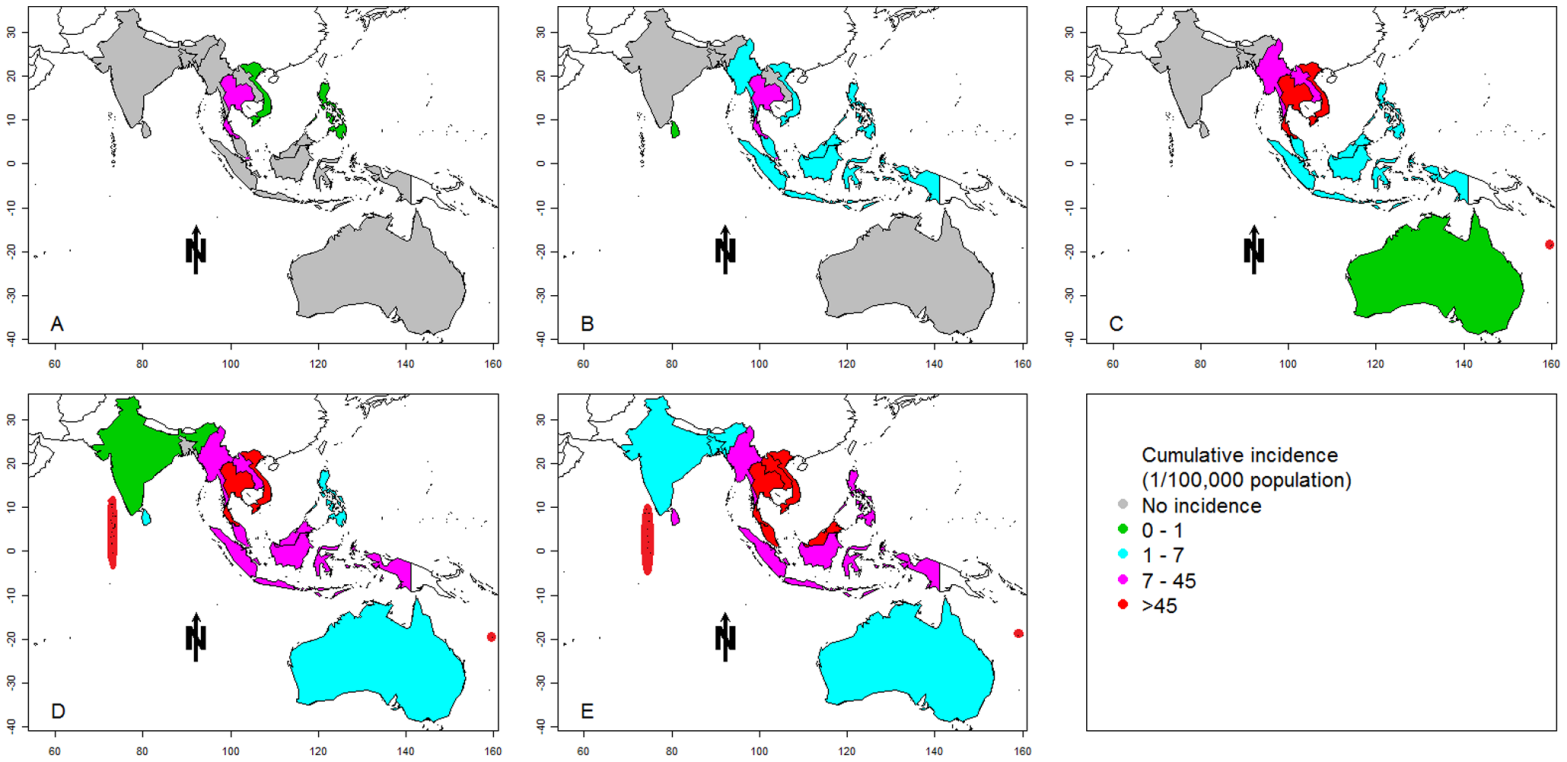
Cumulative incidence of DF in Asia-Pacific countries (A: 1955–1964; B: 1965–1974; C: 1975–1984; D: 1985–1994; E: 1995–2004). The X and Y axes of the map show the longitude and latitude, respectively.

Figure 2 shows that the DF endemic areas had geographically expanded in the Asia-Pacific region over the 50-year study period, and an increasing number of countries were affected over time. On average, at least two new countries experienced outbreaks in each decade (Figure 2). Thailand, Vietnam, Singapore and Philippines were affected in the earlier years of the 1955–1964 period, which suggests that any of these countries could be the origin of DF transmission in the region. Figure 2 also shows that DF expanded mainly in a southward direction in the region. Countries south of Thailand or the Philippines – such as Indonesia, Malaysia, Australia, and other Pacific Islands – have become infected in recent years. The highest DF incidence (2123/100 000 people) was observed in the Cook Islands between 1995 and 2004.

### Space-time Clusters

**Figure 3:**
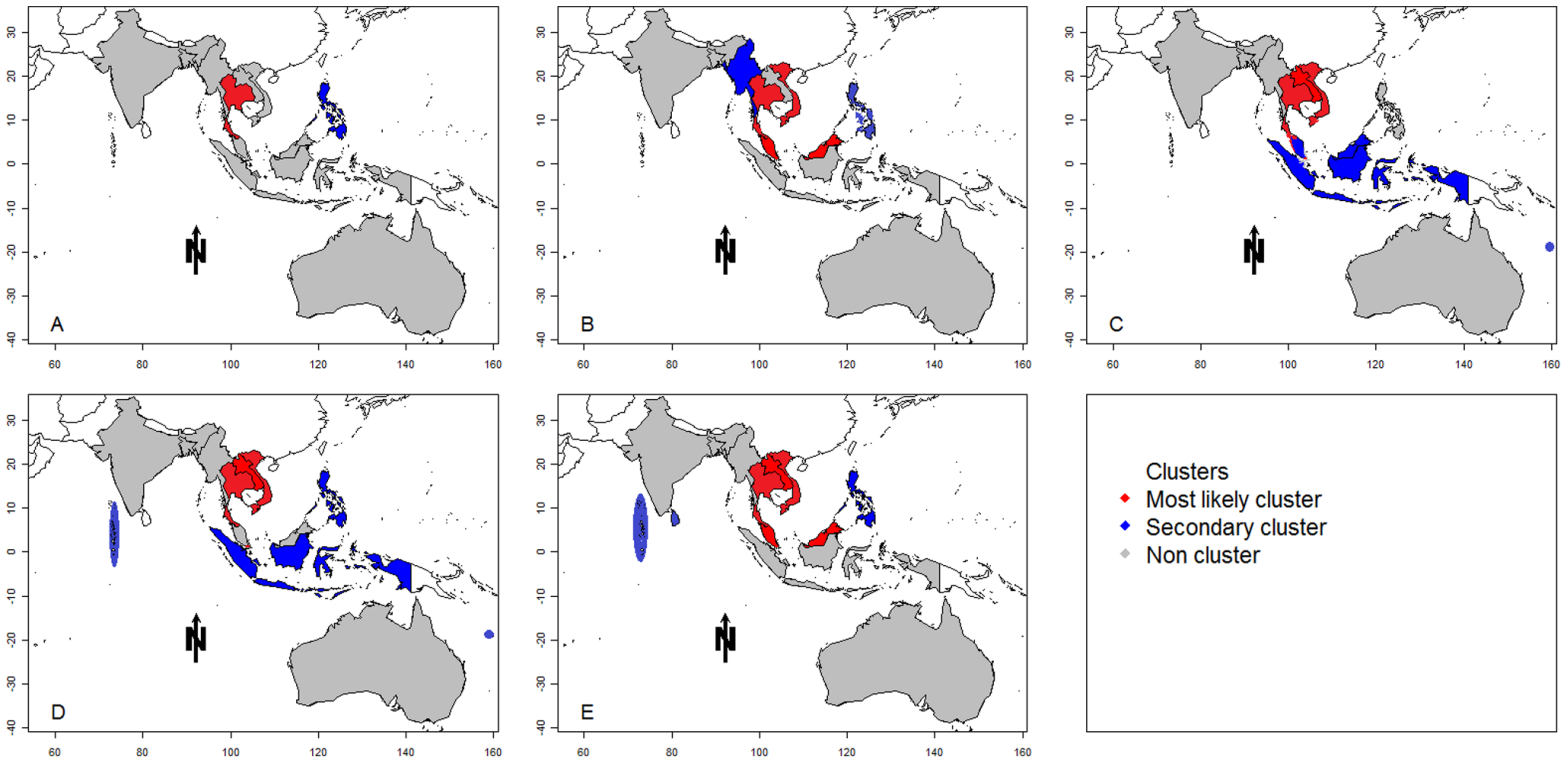
Space-time clusters of DF transmission in the Asia-Pacific region (A: 1955–1964; B: 1965–1974; C: 1975–1984; D: 1985–1994; E: 1995–2004). The X and Y axes of the map show the longitude and latitude, respectively.

Table 2 shows the results of the space-time cluster analysis, stratified in the five periods. Using a maximum cluster size of 15% of the population at risk, SaTScan identified Thailand (RR = 96.13) as the most likely cluster, and the Philippines (RR = 5.96) as the secondary cluster from 1955 to 1964. The most likely cluster detected during the 1965–1974 period covered four countries Singapore, Malaysia, Thailand and Vietnam within a radius of 1711.16 km. Thus, the DF cluster areas substantially increased from 1965 to 1974 compared to the previous ten years, and this trend continued in the following years. In the most recent decade of the study (1995–2004), eight countries were identified as statistically significant DF clusters. The most likely clusters include Singapore, Malaysia, Thailand, Vietnam and Laos (radius = 1872.04 km, RR = 13.02). Overall, it was observed that the DF cluster areas in the Asia-Pacific region had expanded over time (Figure 3).

**Table 2:** Space-time clusters of DF transmission in the Asia-Pacific region, 1955–2004.

Cluster	Countries	Radius (km)	Time frame	No. Obs.	No. Exp.	Relative risk	LLR*
**1955–1964**							
1†	Thailand	0	1960/1/1 to 1964/12/31	18337	527.21	96.13	54814.34
2	Philippines	0	1960/1/1 to 1964/12/31	3092	571.13	5.95	2819.03
**1965–1974**							
1†	Singapore, Malaysia,Thailand, Vietnam	1711.16	1971/1/1 to 1974/12/31	76393	6143.11	24.05	142940.013
2	Philippines	0	1966/1/1 to 1966/12/31	9384	528.09	18.88	18411.19
3	Myanmar	847.81	1974/1/1 to 1974/12/31	2477	1755.02	1.42	133.25
**1975–1984**							
1†	Vietnam, Laos, Thailand	812.13	1980/1/1 to 1984/12/31	510942	38105.18	31.06	1024627.77
2	Cook Islands	0	1980/1/1 to 1980/12/31	357	1.31	273.01	1646.82
3	Malaysia	0	1982/1/1 to 1982/12/31	3052	1114.03	2.75	1140.04
4	Indonesia	0	1983/1/1 to 1984/12/31	26585	23294.07	1.15	228.63
**1985–1994**							
1†	Vietnam, Laos, Thailand	812.13	1987/1/1 to 1991/12/31	1034416	79277	27.18	2009818.49
2	Cook Islands	0	1991/1/1 to 1991/12/31	1776	2.54	699.17	9858.32
3	Indonesia	0	1988/1/1 to 1988/12/31	44573	23048	1.96	7995.79
4	Maldives	0	1988/1/1 to 1988/12/31	2054	30.41	67.61	6630.25
5	Philippines	0	1991/1/1 to 1991/12/31	11317	8290	1.37	497.68
**1995–2004**							
1†	Singapore, Malaysia,Thailand, Vietnam, Laos	1872.04	1995/1/1 to 1998/12/31	852301	95356.45	13.02	1243215.57
2	Philippines	0	2001/1/1 to 2004/12/31	94651	45564.40	2.12	21013.62
3	Sri Lanka, Maldives	983.29	2002/1/1 to 2004/12/31	29895	8771.22	3.44	15623.32

No. Obs, number of observed cases; No. Exp, number of expected cases; LLR, Log -likelihood Ratio.

*P<0.05;

† Most likely cluster.

## Discussion

The results of this study indicate that the geographical range of DF transmission in the Asia-Pacific region expanded during the 1955–2004 period. On average, at least two countries joined the DF cluster areas every ten years. There are many factors that could be responsible for the geographic spread of DF in the region during the 20^th^ century; for example, unprecedented population growth, unplanned urbanization, a lack of effective vector control, and international travel [23], [24]. The movement of troops and materials during World War II might also have played a crucial role in the dissemination of the *Aedes* mosquitoes and the virus [24], [25]. Another possibly important factor was the enormous economic growth in Southeast Asia after World War II [6], [26]. This economic growth led to unplanned urbanization, which resulted in millions of people living in shanty towns with inadequate housing, water supplies and waste management facilities. These overcrowded communities with large mosquito populations create ideal conditions for DF transmission [27]–[29]. In addition, the increased use of modern transportation resulting from globalization is responsible for the importation of the dengue virus through both viremic individuals and the dispersal of exotic mosquitoes into new areas. It has been suggested, for example, that *Aedes albopictus* was introduced into many Pacific islands through modern container ships [23], [30].

Global climate change is also suggested as an important factor in the extent of the expansion of DF in Asia [31]. A recent study in Taiwan shows that urbanization and increased temperature due to climate change are the most important risk factors for its transmission [17]. Climatic factors including temperature, rainfall and humidity have direct and indirect impacts on mosquito survival, their life span and reproductive rate. This, in turn, can influence the geographic distribution of the virus and vectors [32]. Indeed, an association between DF incidence and rainfall has been reported in many countries of the Asia-Pacific region where outbreaks usually coincide with the rainy season [33]. This is because rainfall can potentially increase the number of mosquito breeding sites which, in turn, increases the chance of DF transmission [34].

Thailand, Vietnam, Laos, Singapore and Malaysia are identified as the most likely DF clusters in the most recent years of the study (1995–2004). DF transmission in these areas follows a cyclical pattern, with the highest incidence in the hot and rainy seasons from May to October [35], [36]. We also know that DF infection in travelers varies according to destination, season of travel, duration of stay and epidemic activity. Therefore, travelers to these cluster countries need to take precautions, such as avoiding the monsoon season and shortening the duration of their stay if a DF outbreak occurs. This awareness could significantly reduce the risk of DF transmission to non-endemic areas.

Our results also suggest that the geographic spread of DF in the Asia-Pacific region could have originated in the Philippines or Thailand, as these two countries were identified as DF clusters as early as 1960. Many other studies also suggest that the Philippines or Thailand could be the origin of DF transmission in Asia [7]. Historically, the first severe DF outbreak occurred in Manila (Philippines) in 1953, and the second outbreak was in Bangkok (Thailand) [24], [37].

In the Asia-Pacific region, DF spread mainly in a southerly direction. Global climate change might explain this southward expansion to some extent. In the past 100 years, for example, mean surface temperature has increased by 0.3–0.8°C across the continent [38]. This could have created climatic conditions suitable for dengue mosquito vector, and facilitated its transmission in the region [26], [31], [39]. A southward spread of DF was also observed in Argentina and Australia [40]–[42]. However, this supposed southward spread has not yet been verified [43].

This study has several strengths. Most importantly, it is the first empirical study to explore the spatiotemporal pattern of DF transmission in the Asia-Pacific region. DF data from 16 countries over a period of 50 years were used in the study. It indicates the necessity for future research to assess important determinants of DF emergence and its rapid geographic spread in the region. It also suggests the importance of exploring DF transmission patterns within specific countries.

The main limitation of this study is the low resolutions of the DF dataset, which only includes annual DF data. Higher resolution could be achieved by monthly or weekly data, which would show the seasonal variations in transmission. However, WHO DengueNet does not provide this information for most countries. Another limitation is the lack of available data from some DF endemic countries in the region, such as Taiwan and China. Inclusion of these data could increase the DF cluster area, and thus help to verify the southward expansion of DF in this region. A limitation also arises from quality issues with the WHO DengueNet data. For example, under-reporting is possible when countries do not report DF outbreak information for years. This information gap can bias study results. Over reporting is also possible, as some countries use only clinical diagnosis rather than serological diagnosis; the latter cannot differentiate DF from other diseases such as chikungunya.

In summary, this study determined that the spatial and temporal distribution of DF in the Asia-Pacific region increased over the 50-year study period. Social, ecological and demographic changes that have occurred in recent years are thought to be responsible for the geographic spread of the disease. Global climate change can also contribute to this spread. Thailand, Vietnam, Laos, Singapore and Malaysia are identified as the most likely clusters for DF in the Asia-Pacific region. This new knowledge can contribute to the improvement of DF prevention and control strategies in the region by prioritizing control efforts and directing them where they are most needed.
